# Downregulation of α-Melanocyte-Stimulating Hormone-Induced Activation of the Pax3-MITF-Tyrosinase Axis by Sorghum Ethanolic Extract in B16F10 Melanoma Cells

**DOI:** 10.3390/ijms19061640

**Published:** 2018-06-01

**Authors:** Da Hyun Lee, Sung Shin Ahn, Jung-Bong Kim, Yoongho Lim, Young Han Lee, Soon Young Shin

**Affiliations:** 1 Department of Biological Sciences, Sanghuh College of Life Sciences, Konkuk University, Seoul 05029, Korea; ldgyl@naver.com (D.H.L.); wendy7130@naver.com (S.S.A.); yhlee58@konkuk.ac.kr (Y.H.L.); 2 Functional Food and Nutrition Division, National Institute of Agricultural Sciences, Rural Development Administration, Jeonju 55365, Korea; jungbkim@korea.kr; 3 Division of Bioscience and Biotechnology, BMIC, Konkuk University, Seoul 05029, Korea; yoongho@konkuk.ac.kr; 4 Cancer and Metabolism Institute, Konkuk University, Seoul 05029, Korea

**Keywords:** sorghum, melanogenesis, α-melanocyte stimulating hormone, tyrosinase, microphthalmia-associated transcription factor, paired box gene 3

## Abstract

Ultraviolet irradiation-induced hyperpigmentation of the skin is associated with excessive melanin production in melanocytes. Tyrosinase (TYR) is a key enzyme catalyzing the rate-limiting step in melanogenesis. TYR expression is controlled by microphthalmia-associated transcription factor (MITF) expression. Sorghum is a cereal crop widely used in a variety of foods worldwide. Sorghum contains many bioactive compounds and is beneficial to human health. However, the effects of sorghum in anti-melanogenesis have not been well characterized. In this study, the biological activity of sorghum ethanolic extract (SEE) on α-melanocyte-stimulating hormone (α-MSH)-induced TYR expression was evaluated in B16F10 melanoma cells. SEE attenuated α-MSH-induced *TYR* gene promoter activity through the downregulation of the transcription factor MITF. We found that paired box gene 3 (Pax3) contributes to the maximal induction of *MITF* gene promoter activity. Further analysis demonstrated that SEE inhibited α-MSH-induced Pax3 expression. The collective results indicate that SEE attenuates α-MSH-induced TYR expression through the suppression of Pax3-mediated *MITF* gene promoter activity. Targeting the Pax3-MITF axis pathway could be considered a potential strategy to increase the efficacy of anti-melanogenesis.

## 1. Introduction

Melanin in the skin is a natural pigment induced by ultraviolet (UV) radiation of solar light. It plays a role in absorbing UV rays to maintain body temperature and to protect skin cells against UV-induced DNA damage. However, excessive exposure to UV light or aging of the skin may increase abnormal melanin pigmentation of the face, armpits, and hips, resulting in dark spots, such as facial spots and freckles.

Skin pigmentation largely depends on the deposition of melanin produced from melanocytes located in the epidermis, in its basal layer over the base membrane. When exposed to UV radiation, in particular, UV-B light, keratinocytes secrete α-melanocyte-stimulating hormone (α-MSH), an endogenous peptide hormone of the melanocortin family with an acetyltridecapeptide structure [1]. α-MSH binds to the melanocortin-1 receptor (MC1R) on melanocytes. MC21R is a member of a family of G protein-coupled receptors. Activated MC1R triggers the stimulation of adenylate cyclase and subsequently increases the cyclic adenosine monophosphate (cAMP) level, leading to the activation of cAMP-dependent protein kinase (PKA), which in turn phosphorylates the transcription factor cAMP response element-binding protein (CREB) [2,3]. The phosphorylated CREB becomes active and binds to the cAMP response element (CRE) site within the promoter region of the microphthalmia-associated transcription factor *(MITF)* gene, a master regulator of melanogenesis [2]. The accumulated MITF protein activates the transcription of several genes involved in melanin biosynthesis, including tyrosinase (TYR), tyrosinase-related protein 1 (TRP1), and tyrosinase-related protein 2 (TRP2, also known as dopachrome tautomerase) [4]. TYR is a membrane-bound enzyme located on the melanosome, which catalyzes the oxidation of tyrosine to cAMP response element (CRE) [5,6]. DOPA-quinone is then converted to the melanin polymer by TRP1 and TRP2 [7]. Mutation of the *TYR* gene is associated with albinism phenotypes in humans [8] and mice [9]. Mutation of the mouse *MITF* gene also leads to hypopigmentation [10]. Therefore, when TYR or MITF activity is reduced, the melanin biosynthesis process is interrupted, which can reduce skin hyperpigmentation. In the cosmetics industry, various TYR inhibitors are widely used in skin depigmentation [11].

Sorghum (*Sorghum bicolor* L. Moench) is an important cereal crop that is used in a variety of foods in various parts of the world. Sorghum is a gluten-free grain [12] and a rich source of nutrients, including proteins, lipids, niacin, iron, vitamin B_1_, and vitamin B_12_, as well as various bioactive compounds, including phenolic acids, flavonoids, and phytosterols [13,14]. Sorghum phytochemicals have a broad range of human health benefits, including cholesterol-lowering effects, antioxidant capacity, and cancer prevention [14,15]. Phenolic and polyphenolic compounds capable of inhibiting the production of melanin include hydroquinone, arbutin, kojic acid, glabridin, diosmetin, resveratrol, and quercetin [16,17,18,19,20,21,22]. Sorghum contains various phenolic acids and flavonoids [13]. However, little is known regarding the mechanism of Sorghum inn the inhibition of tyrosinase gene expression.

This study evaluated the potential of sorghum ethanolic extract (SEE) as an anti-melanogenic agent. Inhibitory effects of the SEE on α-MSH-induced melanogenesis were investigated using reverse transcription-polymerase chain reaction (RT-PCR), quantitative real-time PCR (qPCR), and gene promoter reporter assay in murine B16F10 melanoma cells. Additionally, we explored the molecular mechanisms involved in the regulation of *TYR* and *MITF* gene expression in response to SEE. The results demonstrate that SEE prevents the activation of the α-MSH−MITF−TYR axis by attenuating Pax3-mediated MITF expression in B16F10 melanoma cells.

## 2. Results and Discussion

### 2.1. SEE Inhibits α-MSH-Induced TYR Expression

We first examined whether SEE is cytotoxic to B16F10 melanoma cells. Treatment with SEE did not show any cytotoxicity at concentrations up to 100 μg/mL, as revealed by short-term (24 h) cell viability assay (Supplemental Figure S1). We used 50 and 100 μg/mL concentrations for subsequent experiments.

**Figure 1:**
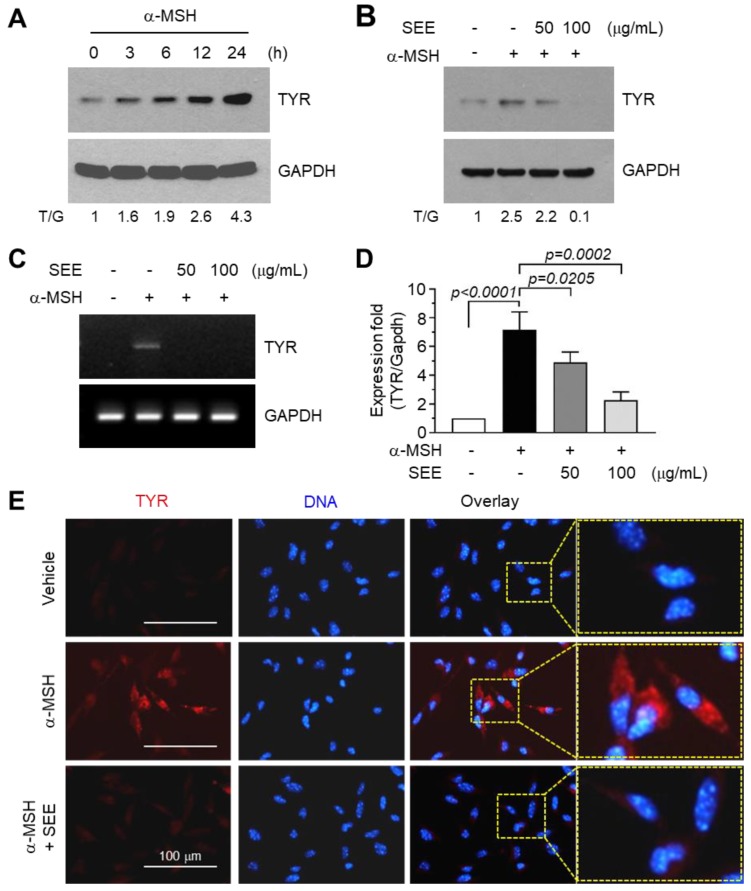
Effect of sorghum ethanolic extract (SEE) on the suppression of alpha-melanocyte-stimulating hormone (α-MSH)-induced tyrosinase (TYR) expression. (**A**) B16F10 cells were treated with 100 nM α-MSH for various times (0–24 h) and cell lysates were subjected to immunoblotting using antibodies against TYR. The glyceraldehyde 3-phosphate dehydrogenase (GAPDH) level was examined as an internal control. T/G, tyrosinase/GAPDH (**B**) B16F10 cells were treated with either vehicle (DMSO) or SEE (50 and 100 μg/mL) for 30 min, followed by stimulation with 100 nM α-MSH. After 12 h, cell lysates were prepared, and immunoblotting was performed using antibodies against TYR. The GAPDH level was examined as an internal control. The intensity of the bands was quantified using ImageJ and the relative TYR intensity was normalized to that of GAPDH and visualized in the blot. T/G, TYR/GAPDH. (**C**,**D**) B16F10 cells were treated with either vehicle (DMSO) or SEE (50 and 100 μg/mL) for 30 min, followed by stimulation with 100 nM α-MSH. After 6 h, total RNA was isolated and TYR mRNA was measured by RT-PCR (**C**) and quantitative real-time PCR (**D**). The GAPDH mRNA level was examined as an internal control. (**E**) B16F10 cells were treated with vehicle (DMSO) or SEE (50 μg/mL) in the absence or presence of 100 nM α-MSH. After 12 h, the cells were fixed and incubated with antibodies against TYR for 2 h, followed by incubation with Alexa Fluor 555-conjugated (red signal) secondary antibodies for 30 min. Nuclear DNA was stained with 1 μg/mL Hoechst 33258 for 10 min (blue signal). The dotted box indicates the region of higher magnification in images.

Upon UV exposure, skin keratinocytes secrete α-MSH, which stimulates TYR expression in melanocytes [3]. We confirmed a time-dependent increase in the amount of TYR protein upon α-MSH stimulation in B16F10 melanoma cells (Figure 1A). The basal level of TYR protein in the vehicle-treated control was probably due to the melanogenic effect of the Dulbecco's modified Eagle's medium (DMEM) medium itself (Supplemental Figure S2) [23]. To evaluate the effect of SEE on TYR expression, B16F10 cells were pre-treated with SEE before stimulation with α-MSH, and TYR protein levels were examined using immunoblot analysis. Pre-treatment with SEE at the 50 and 100 μg/mL concentrations dose-dependently inhibited the α-MSH-induced accumulation of TYR proteins (Figure 1B). RT-PCR analysis shows similar results (Figure 1C). To precisely quantify the effect of SEE on TYR mRNA expression, we carried out quantitative real-time PCR (qPCR) analysis. Figure 1D shows that α-MSH-induced TYR mRNA level decreased in the presence of SEE: α-MSH alone, 7.17 ± 1.23-fold; α-MSH plus 50 μg/mL SEE, 4.9 ± 0.72-fold; α-MSH plus 100 μg/mL SEE, 2.27 ± 0.569-fold, compared to the basal level. Immunofluorescence microscopy also showed that SEE substantially decreased the α-MSH-induced positive staining intensity of TYR proteins (Figure 1E). These data demonstrated that SEE significantly decreased α-MSH-induced TYR expression at the mRNA level without eliciting a cytotoxic effect in B16F10 melanoma cells (all *p* < 0.05, *n* = 3).

### 2.2. SEE Inhibits α-MSH-Induced TYR Promoter Activity through the M-Box Element within the TYR Regulatory Region

**Figure 2:**
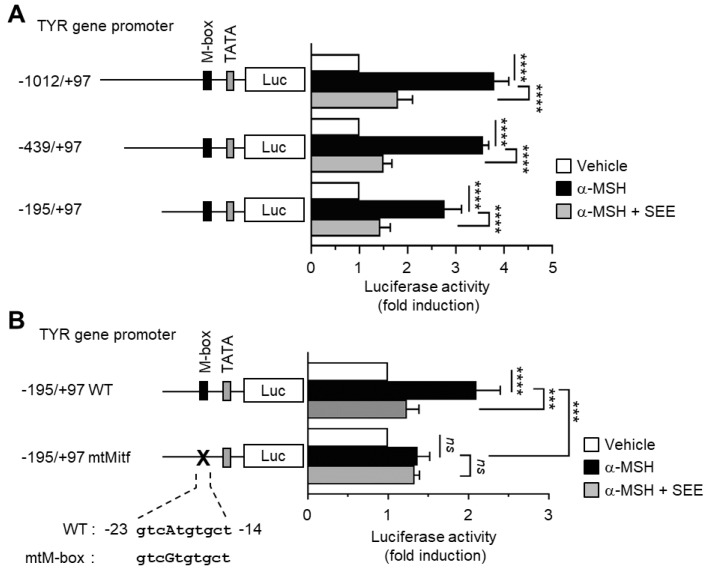
Effect of SEE on the inhibition of α-MSH-induced *TYR* promoter activity. (**A**) B16F10 cells were transfected with 0.2 µg of a series of 5′-deletion constructs of the *TYR* gene promoter reporter plasmids, or (**B**) transfected with 0.2 µg of a wild-type (WT) or site-specific mutant for M-box (mtM-box), derived from the −195/+97 construct. Forty-eight hours later, the cells were treated with either vehicle (DMSO), 100 nM α-MSH, or α-MSH plus 50 μg/mL SEE for 8 h, and luciferase activities were measured. The data shown represent the mean ± SD (*n* = 3). **** *p* < 0.0001; *** *p* = 0.0004; *ns*, not significant.

To determine whether SEE alters *TYR* gene promoter activity, B16F10 cells were transfected with serial deletion constructs (−1012/+97, −439/+97, and −195/+97) of the *TYR* gene promoter reporters. α-MSH stimulation resulted in an increase in *TYR* gene promoter activities, which were significantly reduced (all *p* < 0.0001, *n* = 3) by SEE pre-treatment (Figure 2A), suggesting that SEE downregulates TYR expression at the promoter level. 

MITF is a transcription factor belonging to the family of basic helix-loop-helix leucine zipper proteins. It binds to the highly conserved binding motif (M-box; GTCATGTGCT) in the regulatory region of the *TYR* promoter, and strongly stimulates the melanocyte-specific transcription of the *TYR* gene [24]. We found that a point mutation of the M-box motif (mtM-box; gtcAtgtgct to gtcGtgtgct) at −23/−14 significantly (*p* = 0.0004, *n* = 3) attenuated α-MSH-induced *TYR* gene promoter activation (Figure 2B). SEE did not influence the α-MSH-induced reporter activity in the mtM-box construct, suggesting that a *cis*-acting M-box element may be associated with SEE-induced TYR downregulation. Thus, we considered the possibility that SEE may modulate the transcription factor MITF to reduce the *TYR* transcription due to α-MSH stimulation. 

### 2.3. SEE Downregulates α-MSH-Induced MITF Expression

**Figure 3:**
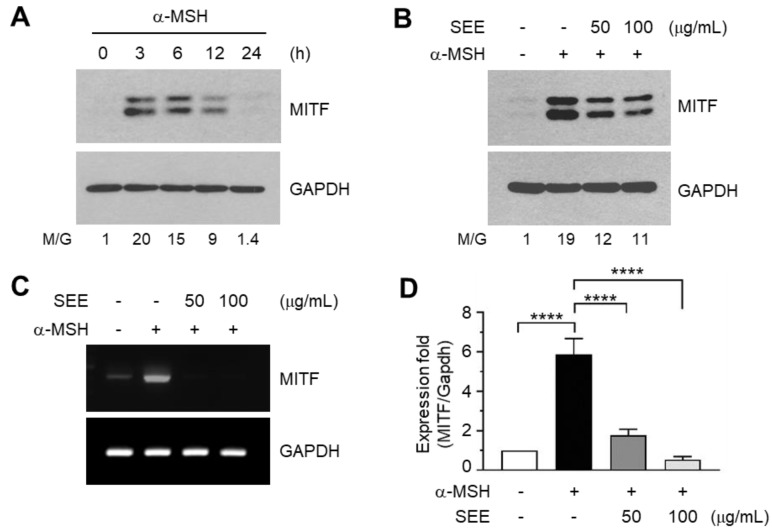
Effect of SEE on the downregulation of α-MSH-induced microphthalmia-associated transcription factor (MITF) expression. (**A**) B16F10 cells were treated with 100 nM α-MSH for various times (0–24 h), and cell lysates were subjected to immunoblotting using antibodies against MITF. GAPDH level was examined as an internal control. The intensity of the bands was quantified using ImageJ and the relative MITF intensity was normalized to that of GAPDH and visualized in the blot. M/G, MITF/GAPDH. (**B**–**D**) B16F10 cells were treated with either vehicle (DMSO), 50 or 100 μg/mL SEE for 30 min, followed by stimulation with 100 nM α-MSH. (B) After 6 h, the cell lysates were subjected to immunoblotting using antibodies against MITF. GAPDH level was examined as an internal control. (**C**,**D**) After 3 h, total RNA was isolated and MITF mRNA was measured by RT-PCR (**C**) and quantitative real-time PCR (**D**). The GAPDH mRNA level was examined as an internal control. The data shown represent the mean ± SD (*n* = 3). **** *p* < 0.0001.

To investigate whether SEE affects MITF expression, we first examined the kinetics of MITF expression in response to α-MSH stimulation in B16F10 cells. The amount of MITF protein reached a peak within 3 h after α-MSH stimulation, with protein levels gradually declining to the basal level thereafter (Figure 3A). α-MSH-induced TYR and MITF expression were observed only in B16F10 cells, not in NIH3T3 fibroblasts, suggesting that the α-MSH response is specific for the cell type (Supplemental Figure S3). Pre-exposure to SEE reduced the α-MSH-induced accumulation of MITF proteins (Figure 3B). RT-PCR analysis also showed that the α-MSH-induced increase in MITF mRNA level was reduced by pre-exposure to SEE (Figure 3C). The relative expression level of mRNA was measured by qPCR. Treatment with α-MSH alone resulted in a 5.87 ± 0.802-fold increase of the MITF mRNA level. However, this was significantly reduced to 1.77 ± 0.306- and 0.533 ± 0.153-fold (all *p* < 0.0001, *n* = 3) by pre-exposure to 50 and 100 μg/mL SEE, respectively (Figure 3D). These results suggest that SEE suppresses α-MSH-induced MITF expression at the mRNA level in B16F10 cells.

### 2.4. SEE Inhibits α-MSH-Induced MITF Gene Promoter Activity

**Figure 4:**
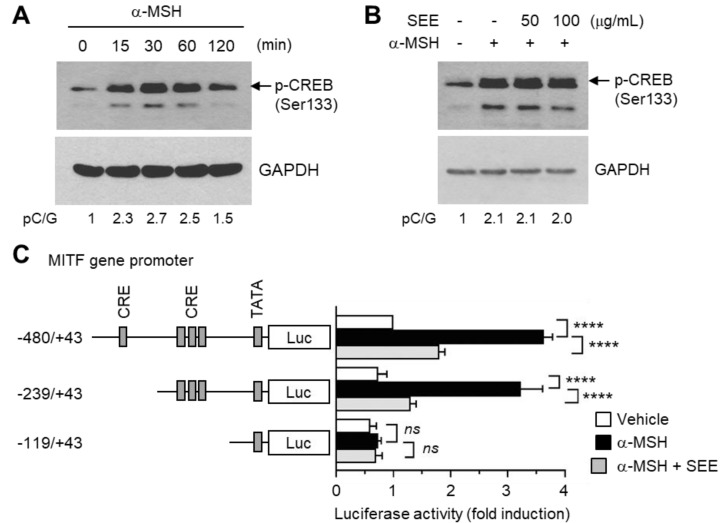
Effect of SEE on the inhibition of α-MSH-induced *MITF* promoter activity. (**A**) B16F10 cells were treated with 100 nM α-MSH for various times (0–120 min), and cell lysates were subjected to immunoblotting using an antibody against phospho-cAMP response element-binding protein (CREB) (Ser133). The glyceraldehyde 3-phosphate dehydrogenase (GAPDH) level was examined as an internal control. (**B**) B16F10 cells were treated with either vehicle (DMSO) or SEE (50 or 100 μg/mL) for 30 min, followed by stimulation with 100 nM α-MSH. After 30 min, cell lysates were subjected to immunoblotting using an antibody against phospho-CREB (Ser133). The GAPDH level was examined as an internal control. The intensity of the bands was quantified using ImageJ and the relative p-CREB intensity was normalized to that of GAPDH and visualized in the blot. pC/G, p-CREB/GAPDH. (**C**) B16F10 cells were transfected with 0.2 µg of a series of 5′-deletion constructs of the *MITF* gene promoter reporter plasmids. Forty-eight hours later, the cells were treated with either vehicle (DMSO), 100 nM α-MSH, or α-MSH plus 50 μg/mL SEE for 8 h, and the luciferase activities were measured. The data shown represent the mean ± SD (*n* = 3). **** *p* < 0.0001; *ns*, not significant.

A previous study demonstrated that the cis-acting cAMP-responsive element (CRE) within the 5′-flanking region of the *MITF* gene contributes to α-MSH-induced *MITF* promoter activity [25]. The CRE-binding protein (CREB) is phosphorylated on serine-133 by PKA upon UV irradiation, and binds to the CRE within the *MITF* promoter [26]. We tested whether α-MSH could stimulate CREB phosphorylation in B16F10 cells. Figure 4A shows that CREB phosphorylation on serine-133 was detected within 15 min after α-MSH stimulation, and peaked at approximately 30 min, after which the phosphorylation gradually decreased to that of unstimulated cells. Unexpectedly, pre-exposure to SEE had no effect on α-MSH-induced CREB phosphorylation at 50 μg/mL concentration and slightly decreased α-MSH-induced CREB phosphorylation at 100 μg/mL (Figure 4B). The results suggest that inhibition of CREB may be not sufficient to explain the inhibitory effect of SEE on the α-MSH-induced MITF mRNA expression. 

The *MITF* gene contains multiple regulatory regions within the 5′-flanking region [4]. To determine whether SEE affects the *MITF* promoter activity and if so, which regulatory region is required for SEE-mediated suppression of the *MITF* promoter action, we generated a series of reporter plasmids containing a 5′-regulatory region of the *MITF* gene promoter (−480/+43, −239/+43, and −119/+43). Luciferase reporter activities of the −480/+43 and −239/+43 constructs were increased by α-MSH stimulation, which was significantly reduced (*p* < 0.0001, *n* = 3) by pre-exposure to SEE (Figure 4C). Further deletion to −120 nt resulted in the loss of both α-MSH and SEE responsiveness. These data suggest that SEE downregulates MITF expression at the promoter level and that the SEE response element responsible for the suppression of the *MITF* transcription is probably localized to the region between −239 and −119.

### 2.5. SEE Downregulates Pax3 Expression to Inhibit α-MSH-Induced MITF Promoter Activity

**Figure 5:**
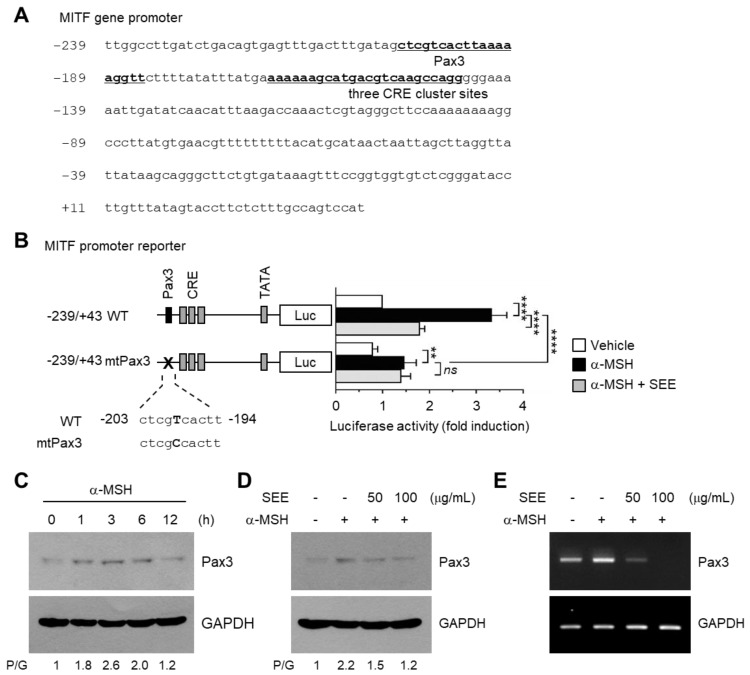
Effect of SEE on the inhibition of α-MSH-induced *MITF* promoter activity via suppression of Pax3. (**A**) DNA sequence of the proximal promoter region of the *MITF* gene. The putative Pax3 and cAMP response element (CRE) cluster sites are underlined. The transcription start site is designated as +1. (**B**) B16F10 cells were transfected with 0.2 µg of a wild-type (WT) or site-specific mutant for Pax3 (mtPax3) derived from −239/+43 construct. Forty-eight hours later, the cells were treated with either vehicle (DMSO), 100 nM α-MSH, or α-MSH plus 50 μg/mL SEE for 8 h, and the luciferase activities were measured. The data shown represent the mean ± SD (*n* = 3). **** *p* < 0.0001; ** *p* = 0.0020; *ns*, not significant. (**C**) B16F10 cells were treated with 100 nM α-MSH for various times (0–12 h), and cell lysates were subjected to immunoblotting using antibodies against Pax3. The GAPDH level was examined as an internal control. The intensity of the bands was quantified using ImageJ and the relative Pax3 intensity was normalized to that of GAPDH and visualized in the blot. P/G, Pax3/GAPDH. (**D**,**E**) B16F10 cells were treated with either vehicle (DMSO) or SEE (50 or 100 μg/mL) for 30 min, followed by stimulation with 100 nM α-MSH for 3 h. Cell lysates were subjected to immunoblotting using an antibody against Pax3 (**D**), and the total RNA was isolated, and Pax3 mRNA was measured by RT-PCR (**E**). The GAPDH level was examined as an internal control.

To identify the SEE response element responsible for the suppression of MITF promoter activity, genomic sequences between −239 and −119 were analyzed using the transcription factor search tool MatInspector (http://www.genomatix.de/). This revealed a consensus binding sequence of Pax3, a transcription factor containing paired-box homeodomain [27], located at −203/−185 within the *MITF* promoter (Figure 5A). Previous studies have reported that mutation of *Pax3* is associated with melanocyte deficiency and hypopigmentation in type I and type II Waardenburg syndrome [28]. In addition, it has been reported that Pax3 binds to the *MITF* promoter and transactivates the *MITF* promoter [4,29]. 

To determine the possible contribution of the Pax3-binding element in α-MSH-induced *MITF* transcription, the core sequence of the Pax3-binding motif spanning −203 to −194 within the −239/+43 construct was mutated from ctcgTcactt to ctcgCcactt (mtPax3) by site-directed mutagenesis (Figure 5B). The mtPax3 construct shows somewhat less promoter activity in response to α-MSH, as compared to the wild-type (WT) construct (from 3.33 ± 0.321 to 1.47 ± 0.252; *p* < 0.0001, *n* = 3), although still leading to a significant increase (*p* = 0.0061, *n* = 3) in α-MSH-induced *MITF* transcription. These data suggest that Pax3 is necessary for the full activation of the *MITF* promoter. Disruption of the Pax3-binding motif had no effect on SEE-mediated inhibition of the *MITF* promoter activity by α-MSH. Therefore, it is possible that SEE may target Pax3 to inhibit *MITF* expression.

To determine the role of Pax3 in SEE suppression of MITF expression, the abundance of α-MSH-induced Pax3 protein level was examined using immunoblotting. The amount of Pax3 protein was rapidly elevated within 1 h, reaching a peak at 3 h, and then slowly decreasing over a period of 6 h after α-MSH stimulation (Figure 5C). We then assessed whether α-MSH-induced Pax3 expression was affected by SEE. Pre-exposure to SEE reduced the α-MSH-induced increase in the level of Pax3 protein in a dose-dependent manner (Figure 5D). The inhibitory effect of SEE on α-MSH-induced Pax3 mRNA expression was confirmed by RT-PCR (Figure 5E). Collectively, these results suggest that SEE attenuates α-MSH-induced TYR expression by suppressing Pax3-mediated activation of the *MITF* gene promoter activity in B16F10 cells. 

**Figure 6:**
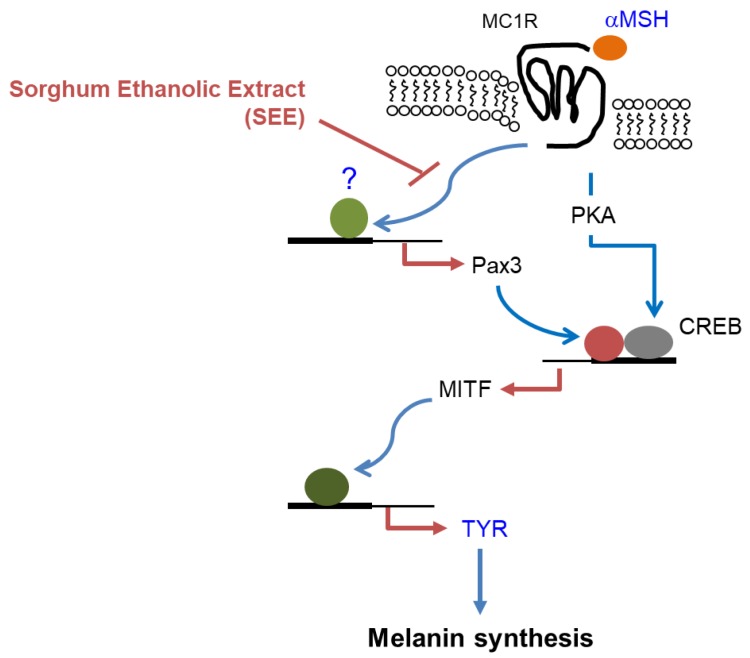
Hypothetical mode of action of SEE on the suppression of α-MSH-induced pigmentation in murine B16F10 melanoma cells. When α-MSH binds to MC1R on the surface of melanocytes, PKA is activated and induces MITF expression via CREB phosphorylation, leading to upregulation of melanogenic enzymes, including tyrosinase. In B16F10 melanoma cells, α-MSH can upregulate the Pax3 expression, which then stimulates MITF gene transcription. SEE inhibits α-MSH-induced Pax3 expression, resulting in the suppression of MITF-mediated TYR expression in B16F10 cells. αMSH, α-melanocyte-stimulating hormone; MC1R, melanocortin 1 receptor; PKA, protein kinase A; CREB, CRE-binding protein; MITF, microphthalmia-associated transcription factor; SEE, sorghum ethanolic extract.

Pax3 participates in the development of the central nervous system, skeletal muscles, and neural crest-derived cell types, including gastrointestinal enteric ganglia and melanocytes, during embryogenesis [27]. Several studies have demonstrated that Pax3 is frequently expressed in the aggregated melanocytic region (nevus) and melanomas [30,31,32], as well as in normal epidermal and follicular melanocytes [33]. Waardenburg syndrome (WS), which is associated with mutations in six genes, including *Pax3*, *MITF*, *SNAI2*, *SOX10*, *EDN3*, and *EDNRB*, is a rare genetic disorder characterized by loss of hearing and changes of pigmentation of skin, hair, and eyes [34,35]. There are four clinical subtypes of WS (type I, II, III, IV), depending on the status of the symptoms. Mutation in *Pax3* is associated with type I and III, and *MITF* mutation is associated with type II [36]. Thus, Pax3 appears to play an important role in melanogenesis. Without UV irradiation, keratinocyte-derived transforming growth factor-β (TGFβ) represses Pax3 expression in melanocyte progenitor cells; however, in response to UV exposure, keratinocytes secrete α-MSH and trigger CREB- and Pax3-mediated MITF expression in melanocytes [37]. In this study, we show that disruption of the Pax3-binding site can abrogate α-MSH-induced *MITF* promoter activity even in the presence of CRE, suggesting that Pax3 is required for α-MSH-induced TYR expression. Our findings demonstrate that the main mechanism of inhibitory action of SEE in α-MSH-induced TYR expression is associated with the downregulation of the Pax3–MITF axis independently of CREB in murine B16F10 melanoma cells. Finally, a proposed hypothetical effect of SEE on the suppression of Pax3–MITF–TYR expression is shown in Figure 6. As MITF is a multifunctional transcription factor that activates the transcription of various genes involved in melanogenesis, melanocyte proliferation, and survival [37], we cannot rule out the possibility that SEE may display multiple properties in addition to the suppression of TYR expression. The molecular pathway that details how SEE inhibits Pax3 expression remains to be explored.

Sorghum contains a variety of phytochemicals that exhibit antioxidant, anticancer, cholesterol-lowering, and anti-inflammatory effects, including tannins, phenolic acids, anthocyanins, phytosterols, and policosanols [13,14,15]. Previous studies have shown that some flavonoids, such as quercetin, fisetin, luteolin, and apigenin, inhibit tyrosinase enzyme activity in the oxidation of l-DOPA [38], while hesperedin, epigallocatechin-3-gallate, and hinokitiol inhibit MITF accumulation [39,40]. SEE contains numerous flavonoids, such as anthocyanin, tannin, apigenin, apigeninidin, naringenin, luteolin, and luteolinidin [41]. However, little is known about the effects of the flavonoids in SEE on downregulating Pax3 expression. Further studies are needed to identify the active ingredients involved in the inhibition of the Pax3-MITF-tyrosinase axis.

## 3. Materials and Methods

### 3.1. Cells and Reagents

Mouse melanoma cell line (B16F10) was obtained from American Type Culture Collection (ATCC, Rockville, MD, USA). The cells were cultured in Dulbecco’s modified Eagle’s medium (DMEM) supplemented with 10% (*v*/*v*) heat-inactivated fetal bovine serum (CellGro/Corning, Manassas, VA, USA). α-MSH was purchased from Sigma-Aldrich (Saint Louis, MO, USA). The firefly and Renilla Dual-Glo™ Luciferase Assay System was obtained from Promega (Madison, WI, USA). 

### 3.2. Preparation of S. bicolor (L.) Moench Ethanolic Extract (SEE)

Sorghum was obtained from an affiliated farm operated by the Rural Development Administration (Chonbuk, Korea), and a voucher herbarium specimen was deposited at Konkuk University, Korea. The seeds of sorghum were dried under a shaded lot, and the dried seeds (1000 g) were ground and subjected to an extraction process thrice with ethanol for three days. The ethanolic extract was evaporated using a rotary evaporator, freeze-dried, and stored at −20 °C until further experiments. The final yield was 47.5 g (4.75%). The lyophilized extracts were dissolved in dimethyl sulfoxide (DMSO) to yield a stock solution concentration of 100 mg/mL.

### 3.3. Cytotoxicity Assay

Cell viability was determined using Cell Counting Kit-8 (CCK-8; Dojindo Molecular Technologies, Gaithersburg, MD, USA) according to the manufacturer’s instructions. Briefly, exponentially growing cells (3 × 10^3^ cells/sample) were exposed to either the vehicle or different concentrations of SEE (0, 25, 50, and 100 μg/mL) for 24 h, followed by the addition of CCK-8 solution containing the water-soluble tetrazolium salt WST-8 (2-(2-methoxy-4-nitrophenyl)-3-(4-nitrophenyl)-5-(2,4-disulfophenyl)-2*H*-tetrazolium, monosodium salt), for an additional 1 h. Absorbance at 450 nm was measured using an Emax Endpoint ELISA Microplate Reader (Molecular Devices, Sunnyvale, CA, USA).

### 3.4. Immunoblot Analysis

Antibodies against MITF (sc-56433) and glyceraldehyde-3-phosphate dehydrogenase (GAPDH; sc-32233) were obtained from Santa Cruz Biotechnology (Dallas, TX, USA), and those against phospho-CREB (Ser133) were obtained from Cell Signaling Technology (Beverly, MA, USA). TYR antibody (ab170905) was purchased from Abcam (Cambridge, MA, USA). Cells were lysed and separated as described previously [42]. The blots were incubated with the primary antibodies for 4 h, followed by horseradish peroxidase-conjugated secondary antibodies for an additional 2 h. Band intensities were quantified using the ImageJ software version 1.52a (http://rsbweb.nih.gov/ij/, National Institute of Health, Bethesda, MD, USA) and normalized to the values for GAPDH. 

### 3.5. Reverse Transcription-PCR and Quantitative Real-Time PCR

Total RNA was extracted using a TRIzol RNA extraction kit (Invitrogen, Carlsbad, CA, USA). First-strand cDNA was synthesized using an iScript cDNA synthesis kit (Bio-Rad, Hercules, CA, USA). PCR conditions were as follows: 94 °C for 5 min, followed by 30 cycles consisting of denaturation at 94 °C (30 s), annealing at 55 °C (30 s), and elongation at 72 °C (1 min). The amplified products were subjected to 1% agarose gel electrophoresis. Quantitative real time-PCR (qPCR) was performed on an iCycler iQ^TM^ system (Bio-Rad) using a TaqMan-iQ^TM^ Supermix kit (Bio-Rad), according to the manufacturer’s recommendations. The RT-PCR primers and TaqMan^TM^ fluorogenic probes were designed by Metabion International (Martinsried, Germany). The gene-specific PCR primers were: forward MITF, 5′-CATGGACTTTCCCTTATCC-3′; reverse MITF, 5′-GTGAGATCCAGAGTTGTC-3′; forward TYR, 5′-CTGGCAGATCATTTGTAG-3′; reverse TYR, 5′-CATGGTTTCCAGGATTAC-3′; forward Pax3, 5′-TCCCATGGTTGCGTCTCTAAG-3′ and reverse Pax3, 5′-CTCCACGTCAGGCGTTGTC-3′; forward GAPDH, 5′-ACCCACTCCTCCACCTTTG-3′; reverse GAPDH, 5′-CTCTTGTGCTGCTGGG-3′. TYR TaqMan probe; 5′-carboxyfluorescein (FAM)-CCTCAGGTGTTCCATCGCATAA-Black Hole Quencher-1 (BHQ-1)-3′; MITF TaqMan probe; 5′-FAM-CTCTGCTCGCCTGATCTGGT-BHQ-1-3′; GAPDH TaqMan probe: 5′-Yakima Yellow^TM^-CGTCGCCAGCCGAGCCACATCG-BHQ-1-3′.

### 3.6. Immunofluorescence Microscopy

B16F10 cells were plated on coverslips and treated with either vehicle (DMSO) or 100 nM α-MSH in the absence or presence of 50 μg/mL SEE, fixed in 4% (*w*/*v*) paraformaldehyde, and permeabilized using 0.1% (*v*/*v*) Triton X-100, as previously described [43]. Primary antibodies against TYR were added and incubated for 2 h, followed by addition of AlexaFluor-555-conjugated anti-rabbit secondary antibodies yielding red signals (Invitrogen) for 30 min. Nuclear DNA was stained with 1 μg/mL Hoechst 33258 (Sigma-Aldrich). Labeled cells were examined under an EVOSf1^®^ fluorescence microscope (Advanced Microscopy Group, Bothell, WA, USA).

### 3.7. Construction and Mutagenesis of the Gene Promoter Reporters

The construction of the mouse *TYR* gene promoter fragments, −1012/+97, −493 to +3, and −114 to +3 have been described previously [44]. A *MITF* promoter fragment spanning from nucleotides −480 to +43 was synthesized from human genomic DNA (Promega) by PCR using the primers 5′-GACCAGGATGCAAGAAGAG-3′ (forward: −480F) and 5′-TGGACTGGCAAAGAGAAGGT-3′ (reverse: +43R), and then ligated into the pGL4-basic vector (Promega), yielding pMitf-Luc(−480/+43). A 5′-deletion construct of human *MITF* promoter fragments was synthesized by PCR using the pMitf-Luc(−480/+43) plasmid as a template. The forward primer sequences were 5′-TTGGCCTTGATCTGACAGTG-3′ (−239 to +43), and 5′-ACCAAACTCGTAGGGCTTC-3′ (−119 to +43). One reverse primer, +43R, was used to generate a series of deletion constructs. The amplified PCR products were ligated into the pGL4-basic vector, yielding pMitf-Luc(−239/+43) and pMitf-Luc(−119/+43), respectively. Site-specific mutations of the Pax3-binding elements at −203/−185 within the *MITF* promoter were performed with the QuickChange site-directed mutagenesis system (Stratagene, La Jolla, CA, USA) using the pMitf-Luc(−239/+43) plasmid as a template. Primer sequences used to generate point mutations of the MITF-binding site were 5′-CACTTAAAAAGGTTCTTTTATATTTATG-3′ (forward) and 5′-GCGAGCTATCAAAGTCAAACTC-3′ (reverse). Disruption of the Pax-binding motif was designated as mtPax3. The site-directed mutation was verified by DNA sequencing (Macrogen, Seoul, Korea).

### 3.8. Luciferase Promoter Reporter Assay

B16F10 cells were seeded in 12-well plates and transfected with the promoter-reporter constructs using Lipofectamine™ 2000 (Invitrogen Life Technologies, San Diego, CA, USA), according to the manufacturer’s instructions. Luciferase activity was measured as described previously [42]. The relative amount of luciferase activity in the vehicle-treated cells was designated as 1. The luminescence was measured with a dual luminometer (Centro LB960; Berthold Tech, Bad Wildbad, Germany).

### 3.9. Statistical Analysis

Statistical analysis was carried out with one-way analysis of variance (ANOVA) followed by Sidak’s multiple comparisons test using GraphPad Prism version 7.04 software (GraphPad Software Inc., La Jolla, CA, USA). A value of *p* < 0.05 was considered statistically significant.

## 4. Conclusions

TYR is a key enzyme initiating melanin biosynthesis in melanocytes. MITF regulates the transcriptional activation of the *TYR* gene. Pax3 is necessary for the maximal activation of *MITF* gene expression. The major finding of the current study is that SEE attenuates α-MSH-induced TYR expression through the suppression of the CREB-independent, but Pax3-dependent activation of *MITF* gene promoter activity. Targeting the Pax3-MITF axis could be considered a potential strategy to increase the efficacy of anti-melanogenesis.

## Supplementary Materials

Supplementary materials can be found at http://www.mdpi.com/1422-0067/19/6/1640/s1.

## Abbreviations

α-MSH	alpha-melanocyte stimulating hormone
BHQ	Black Hole Quencher
cAMP	cyclic adenosine monophosphate
CRE	cAMP-responsive element
CREB	cAMP response element-binding protein
FAM	carboxyfluorescein
GAPDH	glyceraldehyde 3-phosphate dehydrogenase
Luc	luciferase
MITF	microphthalmia-associated transcription factor
Pax3	paired box gene 3
PKA	cAMP-dependent protein kinase
qPCR	quantitative real-time PCR
RT-PCR	reverse transcription-polymerase chain reaction
SEE	sorghum ethanolic extract
TGFβ	transforming growth factor β
UV	ultraviolet
