# Supplementary material for: Downregulation of α-Melanocyte-Stimulating Hormone-Induced Activation of the Pax3-MITF-Tyrosinase Axis by Sorghum Ethanolic Extract in B16F10 Melanoma Cells
Source: Int J Mol Sci. 2018 Jun 1;19(6):1640. doi: 10.3390/ijms19061640 (PMC6032395; doi:10.3390/ijms19061640)
Supplement: Supplementary file 1 [file ijms-19-01640-s001.zip › Supp Figure S1.pdf]

Supplementary Material

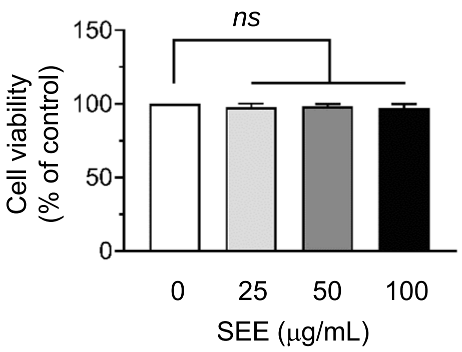

Supplemental Figure S1. B16F10 cells were treated with either vehic le (DMSO; 0) or SEE (25, 50, and 100 μg/mL) for 24 h. Cell viabilities were measured using a Cell Counting Kit-8. Data are presented as me ans ± SD (*n* = 3). *ns* denotes not significant.
