# Supplementary material for: Downregulation of α-Melanocyte-Stimulating Hormone-Induced Activation of the Pax3-MITF-Tyrosinase Axis by Sorghum Ethanolic Extract in B16F10 Melanoma Cells
Source: Int J Mol Sci. 2018 Jun 1;19(6):1640. doi: 10.3390/ijms19061640 (PMC6032395; doi:10.3390/ijms19061640)
Supplement: Supplementary file 1 [file ijms-19-01640-s001.zip › Supp Figure S2.pdf]

Supplementary Material

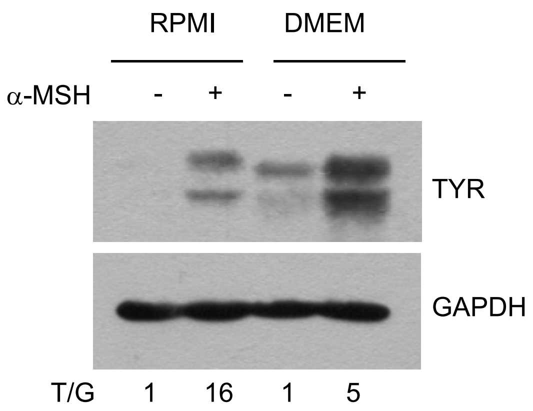

Supplemental Figure S2. B16F10 cells cultured with RPMI or DMEM were treated with either vehicle (DMSO) or 100 nM α-MSH for 24 h. Cell lysates were subjected to immunoblotting using antibody against TYR. The GAPDH level was examined as an internal control. The intensity of band was quantified using ImageJ and the relative TYR intensity was no rmalized to GAPDH and displayed under blot. T/G, TYR/GAPDH.
