# Supplementary material for: Downregulation of α-Melanocyte-Stimulating Hormone-Induced Activation of the Pax3-MITF-Tyrosinase Axis by Sorghum Ethanolic Extract in B16F10 Melanoma Cells
Source: Int J Mol Sci. 2018 Jun 1;19(6):1640. doi: 10.3390/ijms19061640 (PMC6032395; doi:10.3390/ijms19061640)
Supplement: Supplementary file 1 [file ijms-19-01640-s001.zip › Supp Figure S3.pdf]

Supplementary Material

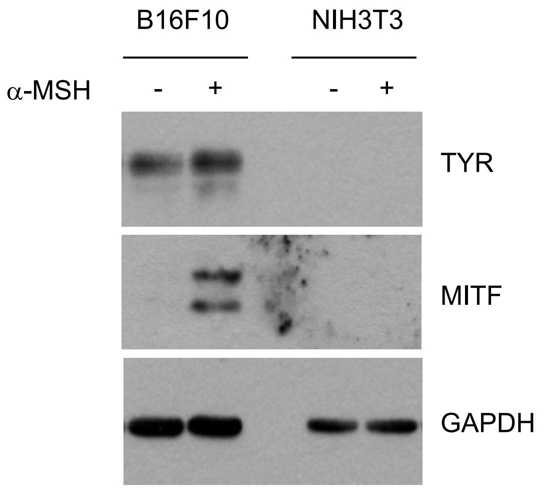

Supplemental Figure S3. B16F10 and NIH3T3 fibroblasts cells were treated with either vehicle (DMSO) or 100 nM α-MSH for 24 h. Cell lysates were subjected to immunoblotting using antibody against TYR or MITF. The GAPDH level was examined as an internal control.
